# Elucidating the Mechanisms of Two Unique Phenomena Governed by Particle-Particle Interaction under DEP: Tumbling Motion of Pearl Chains and Alignment of Ellipsoidal Particles

**DOI:** 10.3390/mi9060279

**Published:** 2018-06-01

**Authors:** Yu Zhao, Jozef Brcka, Jacques Faguet, Guigen Zhang

**Affiliations:** 1 Department of Biomedical Engineering, University of Kentucky, Lexington, KY 40506-0108, USA; yuzhao.bme@uky.edu; 2 Tokyo Electron Technology Center, America, LLC, US-Technology Development Center, Austin, TX 78741, USA; jozef.brcka@us.tel.com (J.B.); jacques.faguet@us.tel.com (J.F.)

**Keywords:** dielectrophoresis (DEP), pearl chain, volumetric polarization and integration (VPI), Arbitrary Lagrangian-Eulerian (ALE), tumbling motion, ellipsoidal particle

## Abstract

Particle-particle interaction plays a crucial role in determining the movement and alignment of particles under dielectrophoresis (DEP). Previous research efforts focus on studying the mechanism governing the alignment of spherical particles with similar sizes in a static condition. Different approaches have been developed to simulate the alignment process of a given number of particles from several up to thousands depending on the applicability of the approaches. However, restricted by the simplification of electric field distribution and use of identical spherical particles, not much new understanding has been gained apart from the most common phenomenon of pearl chain formation. To enhance the understanding of particle-particle interaction, the movement of pearl chains under DEP in a flow condition was studied and a new type of tumbling motion with unknown mechanism was observed. For interactions among non-spherical particles, some preceding works have been done to simulate the alignment of ellipsoidal particles. Yet the modeling results do not match experimental observations. In this paper, the authors applied the newly developed volumetric polarization and integration (VPI) method to elucidate the underlying mechanism for the newly observed movement of pearl chains under DEP in a flow condition and explain the alignment patterns of ellipsoidal particles. The modeling results show satisfactory agreement with experimental observations, which proves the strength of the VPI method in explaining complicated DEP phenomena.

## 1. Introduction

As one of the most studied electro-kinetic phenomena, dielectrophoresis (DEP) has been recognized as having promising potential in manipulating small particles because it can be utilized to move either charged or non-charged particles. Initially, the applications of DEP mainly focused on separating different types of particles. Based on the difference in physical or dielectric property, particles are either guided into separate trajectories or pushed into different regions [1,2,3,4,5]. Lately, due to significant progress made in the field of bio-manufacturing, researchers are paying more attention toward applying the DEP technique to pattern cells based on the induced interactions among cells. For example, cells of different types have been patterned into a liver-like tissue structure with specifically designed electrodes [6,7]. Moreover, cell fusion has been successfully achieved based on the close attachment of cells in gap regions between parallel electrodes [8,9].

To elucidate the governing mechanism for particle alignment, various modeling approaches have been developed, which can be divided into two major classes including the point-dipole approach and the Arbitrary Lagrangian-Eulerian (ALE) moving mesh approach based on the Maxwell stress tensor (MST) method. With the point-dipole approach, some understanding was obtained on how different initial orientations of particles can affect the final alignment pattern [10]. In comparison, the ALE method provides more accurate estimation of the DEP force as well as the hydrodynamic force despite the fact that the number of particles that can be considered is limited. For example, the dynamic movement of several particles (usually two to three) under either alternating current (AC) or direct current (DC) condition have been investigated in detail [11,12].

Most current DEP applications take advantage of the pearl chain structures formed by particles on a planar surface. Normally one peal chain serves as a basic unit for forming large-scale patterns or structures. Nevertheless, the promising potential of application of the DEP technique in bio-manufacturing would be hindered if no other forms of alignment or movement can be generated. To advance the field of DEP and diversify its applications, we conducted experiments coupled with computational modeling to investigate the alignment and movement of particles in fluid flow conditions rather than static conditions. A new form of particle movement termed as the “tumbling motion of pearl chains” has been observed. To explain reasons that cause this unique phenomenon, we adopted two types of modeling approaches called the static force analysis based on volumetric polarization and integration (VPI) method and the coupled ALE-VPI method. The VPI method is our newly developed method which intends to overcome the limitations of both the point-dipole method and the MST method [13]. The point-dipole method neglects the distortion effect of volumetric polarization of particles, which makes it less accurate when the electric field is highly non-uniform. The Maxwell stress tensor method provides the most accurate solution to DEP force quantification. However, the surface integration form often leads to the misconception that the DEP force is a surface force, which results in the incorrect prediction of the movement of particles. Additionally, it fails to correctly calculate the DEP force on non-homogeneous particles due to the surface integration nature, which hinders its application in modeling biologically relevant particles like cells. Therefore, the VPI method has been developed to overcome the disadvantages of the two existing methods. It not only considers the electric field distortion effect but can also accurately quantify the force on non-homogeneous particles with a volumetric integration instead of a surface integration approach, which is used in the Maxwell stress tensor method. It is intended to be used as a universally applicable method for different situations.

In addition, we also investigated the alignment of ellipsoidal particles. For ellipsoidal particles, although some modeling efforts have been made, the modeling results did not capture experimental observations well [14,15]. Considering that a large number of cell types have ellipsoidal shapes, it becomes essential to understand the interaction between ellipsoidal particles if we want to use the DEP technique to pattern cells such as in tissue engineering applications. By employing the VPI method, new insight has been shed in understanding the interaction between ellipsoidal particles.

## 2. Experimental Setup and Observation

### 2.1. Experimental Setup

On the experimental side, the test setup mainly consists of a chip with interdigitated electrodes etched on it as the base. A polyethylene insulation layer was used to cover the chip base and a chamber with both inlet and outlet was built on top of the insulation layer. During the experiments, polystyrene particles suspended in deionised (DI) water were injected into the chamber through the inlet. For electric biasing, an AC potential signal was applied to the interdigitated electrodes in an alternating live/ground manner. The AC signal was generated by a waveform generator (WGF600; FLC Electronics Inc., Partille, Sweden). All optical images were recorded with an inverse optical microscope (Axiovert s100; Zeiss, Oberkochen, Germany).

### 2.2. Experimental Observation

**Figure 1:**
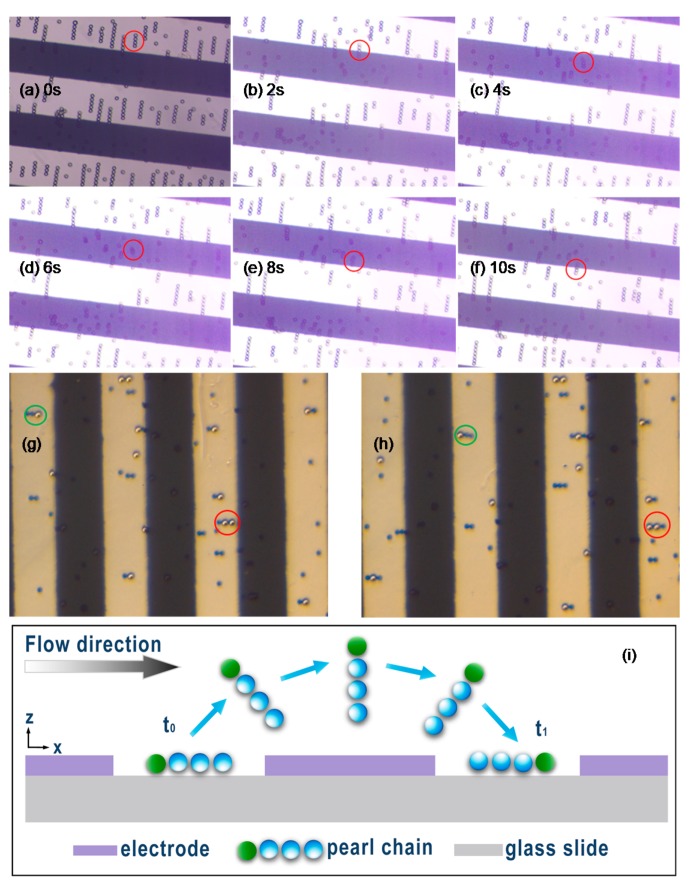
Optical images showing the tumbling motion of particle chains. A chain of four particles circled in red moves with the flow in a gap region (**a**) near the edge of an electrode (**b**), above the electrode off to the upper side (**c**), at the center the electrode (**d**), off the lower side (**e**), and near the other edge of the electrode (**f**). (**g**–**h**) The tumbling motion of particle chains with different sizes and colors. (**i**) Schematic illustration of the tumbling motion of a particle chain. The trailing end of the chain gets lifted up to form an angle with the floor and stand straight at the center of electrodes and, after that, the chain gradually tumbles back as it moves into the next gap region.

10 µm polystyrene particles were mixed in DI water and a small amount of tween 20 was added into the suspension to reduce particle aggregation and adhesion to the substrate. The suspension was then injected into the chamber. Under an AC signal at 20 V_p-p_ (peak-to-peak) and 20 MHz, particles formed pearl-chain structures in the gap regions between electrodes. When a fluid flow perpendicular to the edge of electrodes was introduced to cause the chains to move across electrodes, we observed a very interesting phenomenon in which particle chains initially aligned horizontally in the gap regions moved along with the flow and the chains assumed vertical configurations when they were above the electrodes. This phenomenon can be seen in the images recorded sequentially in time (every 2 s) during experiments (note the highlighted chain in red circles) shown in Figure 1a–f. In a close inspection, it is noted that the change in configurations of the particle chains appears to take a tumbling motion. Since this type of tumbling motion under DEP has not been reported or investigated in past research studies, we repeated the experiment by using particles of different sizes and colors. The sizes of blue and white particles are 10 µm and 15 µm, respectively. Based on these factors, we can ensure that the change in configurations of the particle chains is in a tumbling motion. By tracing the motions and configurations of the same chains of particles in consecutive images in Figure 1g and Figure 1h (e.g., the ones highlighted either in small green circles or in large red circles), we noted that the chains of particles moved along in a clockwise tumbling motion. In this tumbling motion, the trailing particle of a horizontal chain becomes the leading one after assuming a vertical configuration, which is depicted in Figure 1i. The newly observed tumbling motion can be taken advantage of in different aspects. For example, the constantly tumbling pearl chains can be utilized for a solution mixing purpose. It also has promising potential to be used as a driving mechanism for the transport process.

## 3. Theoretical Development

### 3.1. VPI Method (for Spherical and Non-Spherical Particles)

In our previous paper, we have developed a new method based on volumetric polarization to quantify the DEP force exerted on particles [13]. In this paper, we expand this VPI method to quantify the electrostatic potential energy and torque of particles.

A spherical infinitesimal unit inside the particle placed in the electric field is treated as a dipole composed of two point charges with opposite polarity (Q and −Q) and separated by a distance of d (d is the diameter of the spherical particle unit). The potential on the two point charges are $U_{+}$ and $U_{-}$, respectively. The electrostatic potential energy on each point charge can be expressed as $QU_{+}$ and $-QU_{-}$, respectively. By adding these two terms together, we obtain the equation below.

(1) $$w_{p}=QU_{+}-QU_{-}=Q\vec{d}\frac{U_{+}-U_{-}}{\vec{d}}$$

where $w_{p}$ is the electrostatic potential energy of the dipole and $\vec{d}$ is the vector pointing from the negative point charge to the positive point charge. Per the definition of the dipole moment and electric field, Equation (1) can be expressed as:

(2) $$w_{p}=-d\vec{m}\cdot \vec{E}$$

where $d\vec{m}$ is the dipole moment of the particle unit and $\vec{E}$ is the original electric field (as if the particle possesses the same property as the surrounding medium or is not there) at the location of the particle unit.

Based on the previously derived expression of polarization density in terms of the electric field, Equation (2) can be expressed as:

(3) $$w_{p}=-\vec{P}\cdot \vec{E}dV=3\varepsilon_{m}({\vec{E}}_{\mathrm{particle}}-\vec{E})\cdot \vec{E}dV$$

where $\varepsilon_{m}$ is the permittivity of medium and ${\vec{E}}_{\mathrm{particle}}$ is the electric field inside the particle unit. Therefore, the total electrostatic potential energy of the particle ($W_{p}$) is expressed by the equation below.

(4) $$W_{p}=∰3\varepsilon_{m}({\vec{E}}_{\mathrm{particle}}-\vec{E})\cdot \vec{E}dV$$

If the object to be studied is ellipsoidal, the torque needs to be considered besides the electrostatic potential energy. For a particle unit, the torque exerted by the electric field can be derived by the equation below.

(5) $$\vec{t}=\frac{\vec{d}}{2}\times (Q{\vec{E}}_{+})-\frac{\vec{d}}{2}\times (-Q{\vec{E}}_{-})=Q\vec{d}\times \vec{E}=d\vec{m}\times \vec{E}=\vec{P}\times \vec{E}dV=3\varepsilon_{m}(\vec{E}-{\vec{E}}_{\mathrm{particle}})\times \vec{E}dV$$

where ${\vec{E}}_{+}$ and ${\vec{E}}_{-}$ are the electric fields at the positive and negative point charges. The net torque exerted by the electric field on the ellipsoidal particle can also be expressed in summation below.

(6) $$\vec{T}=∰3\varepsilon_{m}(\vec{E}-{\vec{E}}_{\mathrm{particle}})\times \vec{E}dV$$

With Equation (4), the stable orientation of a particle in an electric field can be determined by minimizing the total energy. The torque on the particle will also be examined to ensure the stability of the lowest-energy state. To study the alignment of ellipsoidal particles, we will first validate Equations (4) and (6) by analyzing the orientation of the single ellipsoidal particle and the orientation of a pearl chain formed by spherical particles. We will then investigate the chain configurations of ellipsoidal particles and compare it with experimental observations [14].

### 3.2. Coupled ALE-VPI Method (for Spherical Particle)

To study the dynamic movement of particles in fluid, governing equations for both the fluid flow and solid mechanics are simultaneously solved. For the fluid, the mass and momentum conservation conditions can be expressed by the Navier-Stokes equations below.

(7) $$\rho_{\mathrm{fluid}}\nabla \cdot v_{\mathrm{fluid}}=0$$

(8) $$\rho_{\mathrm{fluid}}\frac{\partial v_{\mathrm{fluid}}}{\partial t}+\rho_{\mathrm{fluid}}(v_{\mathrm{fluid}}\cdot \nabla )v_{\mathrm{fluid}}=\nabla \cdot [-pI+\eta (\nabla v_{\mathrm{fluid}}+{\left(\nabla v_{\mathrm{fluid}}\right)}^{T})]$$

where $v_{\mathrm{fluid}}$ is the velocity of the fluid, $\rho_{\mathrm{fluid}}$ is the density of fluid, p is the hydrodynamic pressure, and η is the dynamic viscosity.

For the particle, the movement is governed by the equations below.

(9) $$\rho_{\mathrm{particle}}\frac{\partial^{2}u_{\mathrm{particle}}}{\partial t^{2}}-\nabla \cdot \sigma =F_{v}$$

where $u_{\mathrm{particle}}$ is the displacement of particle, $\rho_{\mathrm{particle}}$ is the density of particle, σ is the stress tensor, and $F_{v}$ is the volume force density.

At the interface between the particle and fluid, we have the following relationship.

(10) $$v_{\mathrm{fluid}}=\frac{\partial u_{\mathrm{particle}}}{\partial t}$$

(11) $$\sigma \cdot n=[-pI+\mu (\nabla v_{\mathrm{fluid}}+{\left(\nabla v_{\mathrm{fluid}}\right)}^{T})]\cdot n$$

To ensure that multiple particles will not coalesce together, a normal force between two adjacent particles is assigned below.

(12) $${\vec{F}}_{ij}=\left\{ \begin{array}{ll} k\left(q\alpha_{i}-q\alpha_{j}\right)\;\left(\exp\left(\left(2a-d_{ij}\right)/l\right)-1\right) & d_{ij}<=2a\\ 0 & d_{ij}>2a\; \end{array}\right.\;\left(\alpha =x,y,z\right)$$

where $d_{ij}$ represents the distance between particle i and particle j, k is a coefficient, *a* is the radius of particle, l is a small constant value, and qα is the coordinates of particle with α representing one of the three orthogonal directions. To prevent the particle from sinking into the floor, it is also necessary to keep the particle above the bottom floor by applying a proper condition to the particle in the form of a vertical uplifting force to keep the particle afloat.

(13) $${\vec{F}}_{n}=\left\{ \begin{array}{ll} k\left(th+a-qh\right) & qh<th+a\\ 0 & qh\geq th+a \end{array}\right.$$

In this expression, th is the thickness of the insulation layer and qh is the height location of the center of the particle. Equation (13) basically assures that the particle will only experience an uplifting force when it falls (mathematically) below the level of th+a.

Aside from the forces considered above, the particle is also subject to DEP force [13], gravitational force, and buoyancy force. These forces are outlined in the equations below.

(14) $$\vec{F_{\mathrm{dep}}}=∰\left(3\varepsilon_{m}(\vec{E}-{\vec{E}}_{\mathrm{particle}})\cdot \nabla \right)\vec{E}dV$$

(15) $${\vec{F}}_{g}=vol\times \rho_{\mathrm{particle}}\times g$$

(16) $${\vec{F}}_{b}=-vol\times \rho_{\mathrm{fluid}}\times g$$

where vol is the volume of the particle. Since we normally deal with particles with sizes up to tens of microns, the impact of drifting due to diffusion and Brownian motion is regarded as negligible. The particle-fluid-surface system can be simultaneously solved using the ALE method to obtain the positions of particles at each moment.

## 4. Numerical Implementation

### 4.1. Tumbling Motion of Pearl Chains of Particles in a Flow Condition

**Figure 2:**
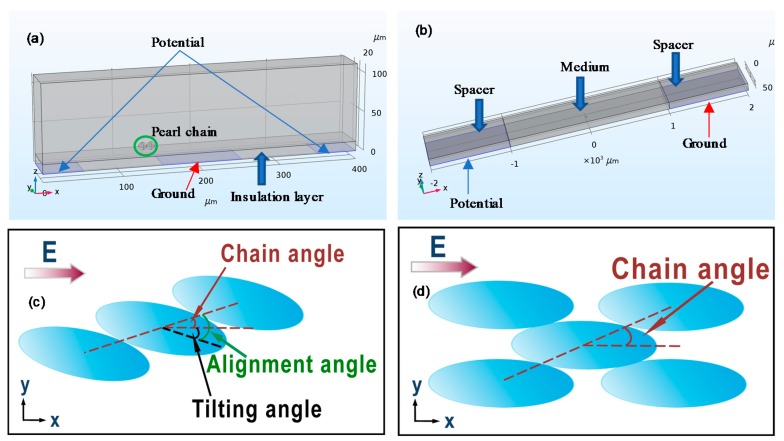
(**a**) Geometry of the 3D model for simulating the tumbling motion of pearl chain in a flow condition. (**b**) Geometry of the 3D model same as the experimental setup of Singh et al. [14] for simulating the alignment of ellipsoidal particles. (**c**) Illustration of ellipsoidal particle alignment in chain configuration. (**d**) Illustration of ellipsoidal particle alignment in stacked configuration.

3D models were built with the same geometry as in experimental setup using COMSOL Multiphysics 5.2a (COMSOL Inc., Stockholm, Sweden), which is shown in Figure 2a. The electrode width and gap distance between electrodes are both set at 100 µm. On top of the electrode is the polyethylene layer (relative permittivity = 2.3) with thickness of 12 µm. To simplify the problem, we only consider pearl chains formed by two particles. To apply the VPI method for static force analysis, two particles in contact are considered in the model. In this case, the orientation of the particle chain as well as the positions of particles can be freely adjusted. The stable orientations of the particle chain at different positions above the electrodes are determined. For the static force analysis method, only the electric current module is solved in the frequency domain study. The two side electrodes are biased by 10 V and the middle electrode is grounded. Periodic boundary conditions are applied to the side walls of the domain and zero charge boundary conditions are applied to the rest walls. For studying the dynamic movement of particles with a coupled ALE-VPI method, two particles in contact are initially placed in the gap region lying on the insulation layer. The same electrical biasing condition found in the static force analysis is applied. A parabolic velocity profile is applied to the inlet and zero pressure is applied to the outlet. By solving the fluid-structure interaction module along with the electric current module in the time dependent study, the movement of particle chains can be simulated. The values of parameters used are listed in Table 1.

### 4.2. Ellipsoidal Particle Alignment

A 3D model was created with the same geometry as in the Singh’s experimental design [14] using COMSOL Multiphysics, which is shown in Figure 2b. In the model, parallel electrodes in a rectangular shape are placed at the bottom with the width set at 1 mm and the length set at 500 µm. The gap distance between two electrodes is 2 mm. On top of the electrode is pure dielectric material with a height of 100 µm to mimic the adhesive spacer used in the experiment. The medium layer with a height of 100 µm occupies the space between the spacers. The parallel electrodes are biased by an AC signal. Periodic boundary conditions are applied to the side walls in the xz plane and zero charge boundary conditions are applied to rest walls. The particle is placed atop the bottom floor in the center of the gap. The electric fields with and without particle presence are first solved with the electric current module and frequency domain study. In Frequency domain, the electric field is expressed as a complex variable. By taking the phase lag between polarization and electric field into consideration, the electrostatic potential energy as well as the torque of the particle can be expressed by the equations below.

(17) $$W_{p}=\frac{1}{2}Re(∰3\varepsilon_{m}({\vec{E}}_{\mathrm{particle}}-\vec{E})\cdot {\vec{E}}^{*}dV)$$

(18) $$\vec{T}=\frac{1}{2}Re(∰3\varepsilon_{m}(\vec{E}-{\vec{E}}_{\mathrm{particle}})\times {\vec{E}}^{*}dV)$$

where ${\vec{E}}^{*}$ is the conjugate of the electric field.

For the case of alignment of multiple particles, it is important to note that the original electric field $\vec{E}$ is different when calculating the electrostatic potential energy and torque. The electrostatic potential energy is a value defined in reference to the original non-distorted electric field. All particles in the system will be assigned with the dielectric property of medium to obtain the original electric field. By contrast, the torque is an absolute value that includes the influence of particle-particle interaction from surrounding particles. Therefore, only the particle to be studied will be assigned with the dielectric property of medium while all other particles are assigned with their intrinsic dielectric properties to obtain the “original” electric field.

#### 4.2.1. Validation of VPI Expressions of Electrostatic Potential Energy and Torque

(1) Orientation of a Single Ellipsoidal Particle

For comparison with the experiment, all ellipsoidal particles with different aspect ratios considered in modeling have the same volume because, in Singh’s experiment, all ellipsoidal particles are made from spherical particles with a diameter of 3 µm. By assuming that the minor axes are of equal length, the length of the major axis and minor axis are $3n^{2/3}$ and $3n^{-1/3}$ µm, respectively, where *n* is the aspect ratio of the ellipsoidal particle. An AC potential signal is applied to the electrodes to create a parallel electric field over the gap region with a root-mean-square (rms) value of 100 V/cm. A single ellipsoidal particle with an aspect ratio of 3 is placed in the center of gap region. For simplification purpose, both the particle and medium are treated as pure dielectric material (conductivity equals zero) with the relative permittivity of medium set at 78.5. Two states of particle polarization are considered here. In the first case, the relative permittivity of the particle is 60 (smaller than that of the medium) and, in the second case, the relative permittivity of the particle is 100 (larger than that of the medium). The orientation of the particle is characterized by the angle between the major axis and the electric field. The particle is parametrically rotated about its geometric center counterclockwise from 0° to 360° with an incremental 30° step. Both electrostatic potential energy and torque of the particle are calculated at each angle.

(2) Orientation of Pearl Chain Formed by Spherical Particles

In this study, we investigate the configuration of particle chains formed by three particles by examining the state of the middle particle. The reason we only study the middle particle is that it can be treated as a representative model for most particles in a long pearl chain. The relative permittivity of the particle and medium are set at 2.5 and 78.5, respectively. The three particles shall always coordinate in a line when the middle particle is in a force equilibrium. In the model, three particles are initially coordinated in a line parallel to the electric field (0°). Considering the symmetrical condition, the chain is parametrically rotated from 0° to 90° counterclockwise with the middle particle fixed. Electrostatic potential energy and the torque of particles are calculated for the middle particle at each angle.

#### 4.2.2. Determination of the Stable Orientation of the Ellipsoidal Particle Chain

To facilitate a comparison with an experimental observation, both the particle and medium are assigned with the same dielectric properties as in the experiment. The conductivity of sodium dodecyl sulfate (SDS) solution with a concentration of 2.5 mM is 2 × 10^−2^ S/m and the surface conductance of polystyrene particles is assumed to be 1.2 nS [16]. The surface conductance is converted to the conductivity of particles based on the relationship for a spherical particle: $K_{\mathrm{total}}=K_{\mathrm{bulk}}+\frac{2K_{s}}{a}$ where $K_{\mathrm{bulk}}$ is the bulk conductivity and $K_{s}$ is the surface conductance. a is the radius of spherical particle (1.5 µm) [17].

We consider three ellipsoidal particles (aspect ratio = 3) initially arranged in a parallel line to the electric field. The two particles on each side rotate in the same direction with the same angle around the geometric center of the middle particle and always maintain a point contact with the middle particle. In this way, the geometric centers of the three particles are always kept in a line, which ensures force equilibrium. The rotating angle of the chain is varied from 0° to 90° with the middle particle fixed in orientation. The electrostatic potential energy and torque are calculated for the middle particle at each angle to determine the stable orientation of the chain. The value of the surface conductance of particles is also varied for studying its effect on the stable orientation of the chain.

## 5. Results and Discussion

### 5.1. Tumbling Motion of Pearl Chains of Particles in a Flow Condition

#### 5.1.1. Static Force Analysis

**Figure 3:**
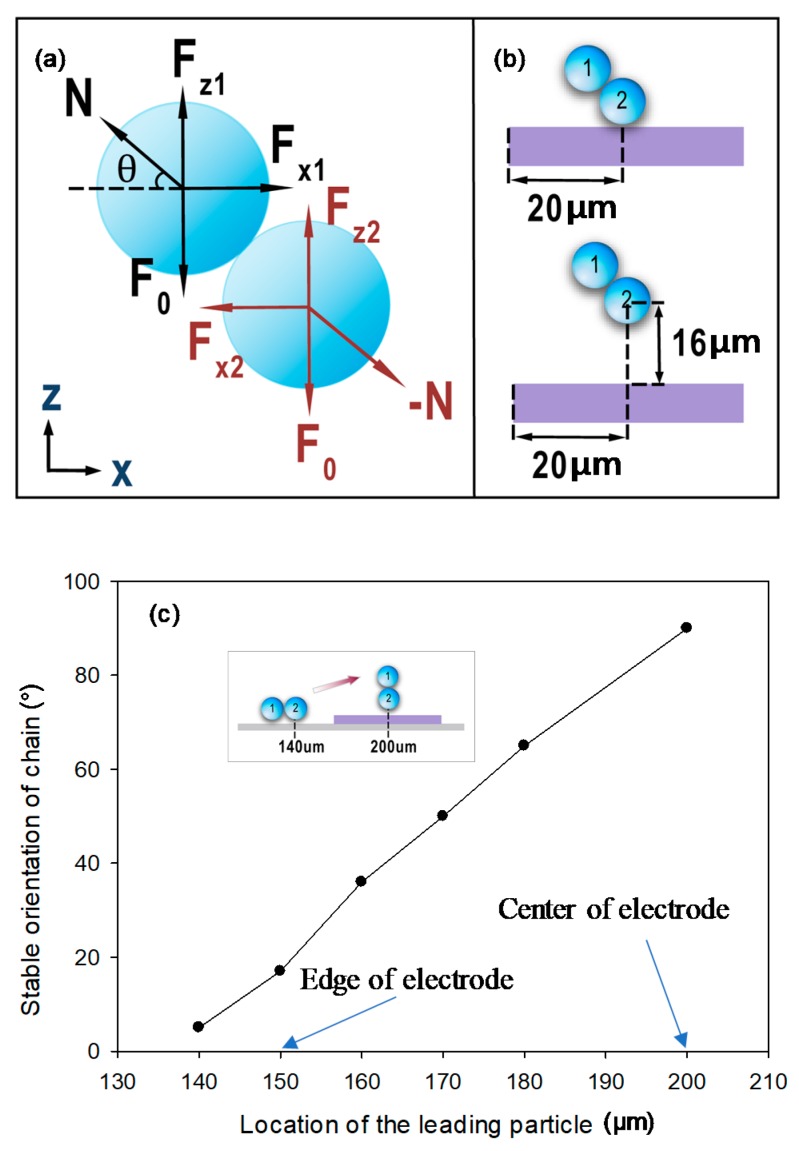
(**a**) A free-body diagram showing two particles in contact where F_0_ equals the sum of gravitational force and buoyancy force, N is normal force, and θ is the angle. (**b**) A schematic showing two-particle chains at different height levels. (**c**) The stable angle formed by the particles with respect to the floor increases as the chain of particles moves from the gap region close to the edge of the electrode to the center of an electrode.

For the tumbling motion to occur, the particles should always remain in a stable contact condition when their positions change. Since the particles will move with the flow, they are assumed to be always in quasi-static condition. Using the free-body diagram shown in Figure 3a where F_x1_, F_z1_, F_x2_ and F_z2_ are the x and z components of the DEP force for particles 1 and 2, respectively, N is the normal contact force, and F_0_ is the sum of other forces (i.e., gravitation + bouyancy) for each particle, we can express the stable contact conditions as (1) F_x1_ – N × cos *θ* = F_x2_ + N × cos *θ* (equilibrium in x direction), (2) F_z1_ + N × sin *θ* = F_0_ and F_z2_ – N × sin *θ* = F_0_ (equilibrium in z direction). F_0_ is estimated to be around 2.57 × 10^−13^ N for 10 µm particles. Therefore, the second condition becomes F_z1_ + N × sin *θ* = F_z2_ – N × sin *θ* = 2.57 × 10^−13^ N. To examine these conditions in detail, we first placed the leading particle of the two-particle chain atop the electrode 20 µm from the left edge on the floor and allowed the trailing particle to assume an oblique position (shown in the upper schematic of Figure 3b). As the oblique angle increases from 0° (horizontal) to 90° (vertical), we obtained the relevant forces, which are listed in Table 2. The normal force N was obtained by assuming an equilibrium condition in the x direction, which was satisfied. Within the angular range from 11.25° to 67.5°, F_z1_ + N × sin *θ* keeps increasing while F_z2_ − N × sin *θ* decreases as the angle increases and they will become equal at some angle between 45° and 56.25°. However, the magnitude is larger than 2.57 × 10^−13^ N. This fact suggests that the two particles will not be in a stable union in this position. However, when the leading particle was placed 16 µm above the floor (shown in a lower schematic of Figure 3b), the situation changed. As listed in Table 3, the condition F_z1_ + N × sin *θ* = F_z2_ − N sin *θ* ≈ 2.57 × 10^−13^ N can be reached when the angle is about 50°, which indicates that the chain of particles will rise slightly and takes this orientation as a stable union. By repeating this procedure with the position of the leading particle set to vary from the gap region close to the edge (140 µm) to the center (200 µm) of the electrode (shown in inset of Figure 3c), we obtained the variation of the stable angle as a function of the leading particle position. Figure 3c shows that the oblique angle of the chain of particles will increase from 5° when the chain is 10 µm away from the edge of the electrode to 90° when the chain is at the center of the electrode. Since the force is symmetrical about the center of the electrode, the chain of particles will tumble back to a horizontal configuration once it moves into the next gap region.

#### 5.1.2. Coupled ALE-VPI Method

**Figure 4:**
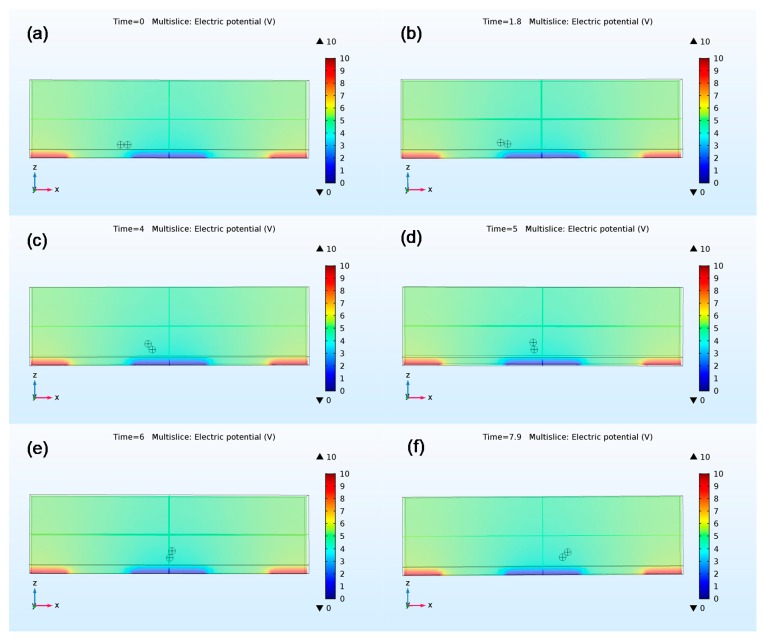
The orientation and position of chains formed by two particles simulated by coupled ALE-VPI method at different time points. (**a**) 0 s, (**b**) 1.8 s, (**c**) 4 s, (**d**) 5 s, (**e**) 6 s, (**f**) 7.9 s. The scale bar represents the potential distribution.

While the coupled ALE-VPI method allows accurate quantification of all relevant forces especially the hydrodynamic force, it has inherent weaknesses. Since the ALE method utilizes the moving mesh technique, the mesh elements will be strongly distorted when the displacement increases, which could lead to a convergence problem. To avoid this problem, the displacement of the particles will be exported and used to reset the initial positions of particles in an incremental iterative process. The average flow velocity is set to be 20 µm/s in the experiment because a higher flow rate makes the process more difficult to converge. Figure 4a–f show the variation of the pearl-chain orientation as the chain moves with flow, which captures the observed tumbling motion for the particle chain. The locations of particle chains at different time points also match experimental recordings. The difference between the results of the coupled ALE-VPI method and static force analysis is that the chain will assume upright configuration before it reaches the center of the electrode in the coupled ALE-VPI method. This is likely due to negligence of the hydrodynamic forces on particles in the static force analysis.

### 5.2. Ellipsoidal Particle Alignment

#### 5.2.1. Validation of VPI Expressions of Electrostatic Potential Energy and Torque

(1) Orientation of a Single Ellipsoidal Particle

**Figure 5:**
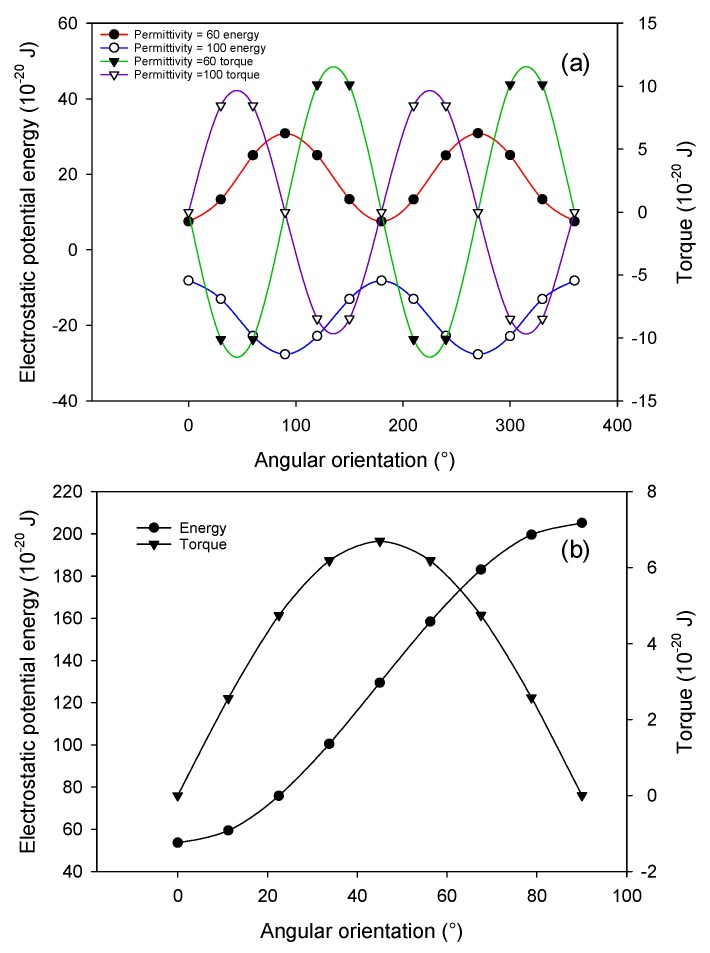
(**a**) Variation of electrostatic potential energy and torque of a single ellipsoidal particle when it rotates for 360°. (**b**) Variation of electrostatic potential energy and torque of the middle spherical particle in three-particle chain when the chain rotates for 90°.

Figure 5a shows the variation of electrostatic potential energy and torque with the orientation of the ellipsoidal particle. When the particle has a relative permittivity of 60, it experiences negative DEP (nDEP). The electrostatic potential energy reaches its minimum at 0° and 180° where the major axis is aligned parallel to the direction of the electric field. The torque is zero when the major axis is either parallel or perpendicular to the electric field direction. By examining the change in torque by introducing a small rotation angle in these orientations, we noted that, only when the major axis is parallel to the electric field, the particle will rotate back to its original orientation. In this case, the electrostatic potential energy and torque are consistent in predicting the stable orientation of the ellipsoidal particle. When the particle has a relative permittivity of 100, it experiences positive DEP (pDEP). Both the electrostatic potential energy and torque predict an orientation of perpendicular alignment. These modeling results agree with both theoretical prediction and the experimental results by Mittal and Furst [18,19] where the titanium oxide ellipsoidal particles align perpendicularly to the electric field at low frequency due to high surface conductance. At high frequency, the particles undergo transition from pDEP to nDEP and start to align parallel to the electric field.

(2) Orientation of Pearl Chain Formed by Spherical Particles

Figure 5b shows the variation of electrostatic potential energy and torque experienced by the middle particle with the orientation of the particle chain. The energy is minimum and the torque is zero when the chain is parallel to the electric field, which suggests that a parallel alignment is a stable configuration for the particle chain formed by spherical particles under nDEP. This conclusion again agrees with experimental observations and confirms the validity of the VPI expressions of energy and torque.

#### 5.2.2. Determination of the Stable Orientation of Ellipsoidal Particle Chain

**Figure 6:**
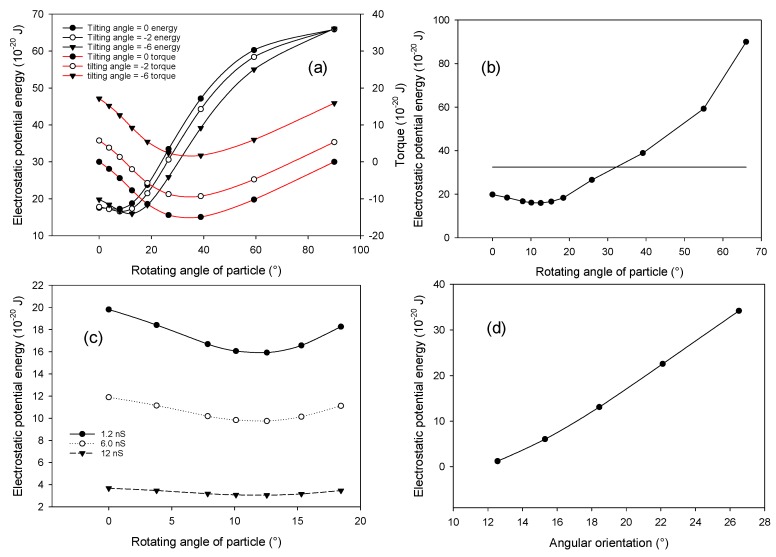
(**a**) Variation of electrostatic potential energy and torque of the middle ellipsoidal particle in three-particle pearl chain as the alignment angle increases from 0° to 90°, (**b**) Variation of electrostatic potential energy of the middle ellipsoidal particle in chain with respect to the alignment angle. The horizontal line represents the minimum energy of a single ellipsoidal particle. (**c**) Particles with different surface conductance have minimum electrostatic potential energy value around the same alignment angle. (**d**) Variation of electrostatic potential energy of the middle ellipsoidal particle in stacked configuration with respect to the diagonal chain angle.

We first define the tilting angle as the angle between the major axis of the ellipsoidal particle and electric field, the chain angle as the angle between the line connecting centers of particles and electric field, and the alignment angle as the angle between the line connecting centers of particles and the major axis of particle, which are shown in Figure 2c. Figure 6a shows that, when the major axes of all three particles are parallel to the electric field (tilting angle equals 0°), the energy reaches its minimum when the alignment angle is slightly larger than 0°, which is different from the case for spherical particles where an energy minimum is obtained when the chain is parallel to the electric field. By examining the torque exerted on the middle particle, we noted that the torque is negative. When the alignment angle further increases, the torque will become even more negative, which indicates that the middle particle will rotate clockwise about its geometric center. This will lead to disassembly of the chain structure. To obtain a stable configuration for the chain, we gradually rotate each ellipsoid particle about its geometric center clockwise (increase the tilting angle) and evaluate the change in energy and torque with respect to the alignment angle. As shown in Figure 6a, the minimum energy will gradually decrease when the tilting angle increases. When the tilting angle reaches 6°, the minimum energy is the lowest. At this minimum energy configuration, the alignment angle is 12.5° and the torque is positive, which ensure contact between particles. Further increase in the tilting angle will lead to an increase of the minimum energy. Therefore, the chain angle is calculated to be 6.5° when the chain is in its lowest minimum energy orientation. Similarly, we can determine the most probable chain angle for ellipsoidal particles with an aspect ratio of 4.3 and 7.6 (Table 4).

Table 4 shows that the most probable chain angles predicted from modeling are smaller than the statistically most probable values measured in experiments. This difference may be attributed to the contact surface topology affecting the stability of the configuration. Since the contact between irregular surfaces is a complex issue, it becomes extremely difficult to consider the actual contact condition in modeling. One may interpret the impact of contact surface topology in an intuitive way. For example, the curvature is larger on the major axis end of the particles, which leads to more concentrated distribution of normal forces and makes the contact less stable. Therefore, ellipsoidal particles favor contact in the small curvature region, which causes the most probable angle to increase.

Figure 6b shows the variation of the electrostatic potential energy with respect to the alignment angle for ellipsoidal particles with an aspect ratio of 3 when the tilting angle is 6°. The horizontal line represents the lowest electrostatic potential energy of a single particle. The results suggest that when the alignment angle is smaller than 32°, the chain is more likely to be stable. Table 5 shows the comparison of modeling and experimental results for all three kinds of particles. The negative value is due to the small tilting angle of the particle with respect to the electric field. Modeling results agree with experimental observation by predicting a narrower distribution of the chain angle as the aspect ratio increases.

Since the exact surface conductance of particles is unknown and likely to affect the stable orientation of chain, three surface conductance values (1.2 nS, 6.0 nS, and 12 nS) are assigned to the ellipsoidal particle with an aspect ratio of 3 (corresponding conductivity of particle equals 1.6 × 10^−3^ S/m, 8 × 10^−3^ S/m, and 1.6 × 10^−2^ S/m). Figure 6c shows the variation of electrostatic potential energy with respect to the alignment angle. For all three cases, the electrostatic potential energy reaches its minimum value at almost the same angle, which suggests that the surface conductance has a minor effect on the stable orientation of the particle chain.

Experimental results show that addition of more particles could lead to a structure shown in Figure 2d [14]. To have such a structure as a favored configuration, the electrostatic potential energy of the middle particle in stacked configuration should be lower than that of the middle particle in a chain. Figure 6d shows the variation of electrostatic potential energy with respect to the chain angle, which is defined as the angle between line connecting centers of the middle particle and one of the surrounding particle and the major axis. Below 12°, the surrounding particles will overlap with each other geometrically. The lowest energy of stacked particles (1.2 × 10^−20^ J) is much lower than the lowest energy of particles in a chain (16 × 10^−20^ J). This suggests that the stacked configuration is more energy-favored than the chain configuration. The modeling results agree with experimental observations when there is a high packing density of particles as well.

The modeling results from our VPI method are then compared with previous modeling results from other modeling methods. In Singh’s work [14], both the particle and medium are treated as pure dielectric material. As the frequency of the AC signal is set at only 10 kHz, the contribution of conductivity dominates permittivity according to the expression of complex permittivity $\varepsilon^{*}=\varepsilon -i\frac{\sigma }{\omega }$, which suggests that the simplification is wrong. In House et al. [15], dynamic modeling based on the MST method was adopted. However, the predicted angles are far different from the experimental measurement and it failed to estimate the range of distribution of the chain angle. Although the influence of contact surface topology has not been accounted for, our modeling results nevertheless provide coherent explanations for the distribution of chain angles and the transition from a chain configuration to a stacked configuration.

## 6. Conclusions

This paper reports a newly observed form of movement of pearl chains in a fluid flow condition under DEP. Computational models based on the VPI method have been developed to elucidate the underlying mechanism for such movement using a static-force analysis approach and dynamic ALE approach. The VPI method has also been used to explain the cause of the unique alignment of ellipsoidal particles from energy and a torque point of view. Satisfactory agreement between modeling results and experimental observations have proven the strength of the VPI method in explaining how particles interact with each other under DEP. It is believed that the new method will provide crucial advancement in understanding complicated DEP phenomena and accelerate new applications of the DEP technique in bio-manufacturing.

## Figures and Tables

**Table 1:** Parameters used in models studying the tumbling motion of pearl chains.

Parameter	Physical Meaning	Value
*k*	Coefficient of normal force	10^−12^ N/µm
*th*	Thickness of insulation layer	12 µm
*l*	A small value to characterize normal force change	1 µm
*ρ*_particle_	The density of particle	1050 kg/m^3^
*ρ*_fluid_	The density of fluid	1000 kg/m^3^
*η*	Dynamic viscosity	10^−3^ Pa·s
*𝜎*_p_	Conductivity of particle	5 × 10^−4^ S/m
*𝜎*_m_	Conductivity of fluid	1.8 × 10^−4^ S/m
*𝜀*_m_	Permittivity of fluid	78.5
*𝜀*_p_	Permittivity of particle	2.5

**Table 2:** Forces on two particles (particle 2 sits atop the electrode 20 µm from the left edge on the floor).

***θ***	**F_x1_ (10^−13^ N)**	**F_x2_ (10^−13^ N)**	**F_z1_ (10^−13^ N)**	**F_z2_ (10^−13^ N)**
0	−3.12	6.50	16.7	3.25 × 10^−2^
11.25	2.40	0.299	14.8	−0.353
22.5	6.49	−4.25	10.8	2.16
33.75	8.28	−6.24	5.72	6.12
45	7.70	−5.63	1.07	10.1
56.25	5.28	−3.00	−2.17	13.0
67.5	1.94	0.683	−3.42	14.0
78.75	−1.33	4.32	−2.60	13.0
***θ***	**F_x1_ − F_x2_ (10^−13^ N)**	**N (10^−13^ N)**	**F_z1_ + N × sin *θ* (10^−13^ N)**	**F_z2_ − N × sin *θ* (10^−13^ N)**
0	−9.61	−4.81	16.7	3.25 × 10^−2^
11.25	2.10	1.07	15.0	−0.562
22.5	10.7	5.81	13.0	−6.56 × 10^-2^
33.75	14.5	8.73	10.6	1.27
45	13.3	9.42	7.73	3.44
56.25	8.28	7.45	4.02	6.79
67.5	1.26	1.64	−1.90	12.5
78.75	−5.64	−14.5	−16.8	27.2

**Table 3:** Forces on two particles (particle 2 sits atop the electrode 20 µm from the left edge 16 µm above the floor).

***θ***	**F_x1_ (10^−13^ N)**	**F_x2_ (10^−13^ N)**	**F_z1_ (10^−13^ N)**	**F_z2_ (10^−13^ N)**
0	−0.700	1.39	6.90	−0.354
11.25	1.78	−1.13	6.22	−0.185
22.5	3.62	−3.00	4.37	1.23
33.75	4.32	−3.68	2.02	3.25
45	3.83	−3.12	−0.134	5.20
47.81	3.56	−2.82	−0.569	5.61
50.63	3.23	−2.46	−0.951	5.97
53.44	2.86	−2.06	−1.28	6.27
***θ***	**F_x1_ − F_x2_ (10^−13^ N)**	**N (10^−13^ N)**	**F_z1_ + N × sin *θ* (10^−13^ N)**	**F_z2_ − N × sin *θ* (10^−13^ N)**
0	−2.09	−1.04	6.90	−0.354
11.25	2.91	1.49	6.51	−0.474
22.5	6.62	3.58	5.74	−0.144
33.75	8.00	4.81	4.69	0.579
45	6.95	4.91	3.34	1.73
47.81	6.38	4.75	2.95	2.09
50.63	5.69	4.49	2.52	2.50
53.44	4.92	4.13	2.04	2.96

**Table 4:** Most probable chain angle from modeling and experimental results.

Aspect ratio	Most Probable Chain Angle (Modeling)	Most Probable Chain Angle (Experiment)
3	6.5°	13°
4.3	5°	12°
7.6	3°	12°

**Table 5:** Distribution of the chain angle from modeling and experimental results.

Aspect ratio	Distribution of Chain Angle (Modeling)	Distribution of Chain Angle (Experiment)
3	−6°–26°	4°–32°
4.3	−4°–20.5°	4°–26°
7.6	−2°–13°	4°–20°
